# Prospective evaluation of a closed-incision negative pressure wound therapy system in kidney transplantation and its association with wound complications

**DOI:** 10.3389/fneph.2024.1352363

**Published:** 2024-02-27

**Authors:** Susanna Lam, Annie Huynh, Tracey Ying, Charbel Sandroussi, David Gracey, Henry C. Pleass, Steve Chadban, Jerome M. Laurence

**Affiliations:** ^1^ Department of Renal Medicine, Royal Prince Alfred Hospital, Sydney, NSW, Australia; ^2^ Transplantation Services, Royal Prince Alfred Hospital, Sydney, NSW, Australia; ^3^ Institute of Academic Surgery, Royal Prince Alfred Hospital, Sydney, NSW, Australia; ^4^ Sydney Medical School, University of Sydney, Sydney, NSW, Australia; ^5^ Charles Perkins Centre, University of Sydney, Sydney, NSW, Australia

**Keywords:** wound complication, kidney transplant, closed incision negative pressure wound therapy, closed incision negative pressure, closed incision management

## Abstract

**Introduction:**

Wound complications can cause considerable morbidity in kidney transplantation. Closed-incision negative pressure wound therapy (ciNPWT) systems have been efficacious in reducing wound complications across surgical specialties. The aims of this study were to evaluate the use of ciNPWT, Prevena™, in kidney transplant recipients and to determine any association with wound complications.

**Material and methods:**

A single-center, prospective observational cohort study was performed in 2018. A total of 30 consecutive kidney transplant recipients deemed at high risk for wound complications received ciNPWT, and the results were compared to those of a historical cohort of subjects who received conventional dressings. Analysis for recipients with obesity and propensity score matching were performed.

**Results:**

In total, 127 subjects were included in the analysis. Of these, 30 received a ciNPWT dressing and were compared with 97 subjects from a non-study historical control group who had conventional dressing. The overall wound complication rate was 21.3% (27/127). There was no reduction in the rate of wound complications with ciNPWT when compared with conventional dressing [23.3% (7/30) and 20.6% (20/97), respectively, *p* = 0.75]. In the obese subset (BMI ≥30 kg/m^2^), there was no significant reduction in wound complications [31.1% (5/16) and 36.8% (7/19), respectively, *p* = 0.73]. Propensity score matching yielded 26 matched pairs with equivalent rates of wound complications (23.1%, 6/26).

**Conclusion:**

This is the first reported cohort study evaluating the use of ciNPWT in kidney transplantation. While ciNPWT is safe and well tolerated, it is not associated with a statistically significant reduction in wound complications when compared to conventional dressing. The findings from this study will be used to inform future studies associated with ciNPWT in kidney transplantation.

## 1 Introduction

Wound complications, including surgical site infections (SSIs) and wound dehiscence, affect between 3% and 27% of kidney transplant recipients (1–4). Risk factors include obesity, diabetes mellitus, age, and immunosuppression (1, 2, 4). SSIs are associated with delayed graft function (DGF) and inferior graft survival, while deep/organ-space infections can lead to graft loss (2, 5, 6). In our unit, the wound complication rate was historically 7.7% (7). However, management can be complex and, in our experience, may include multistage debridement, abdominal wall reconstruction, delayed wound closure with open negative pressure wound therapy (NPWT), and prolonged wound healing (8). This leads to extended hospitalization, increased costs, and negative patient experiences (9–13).

Therefore, a proactive approach to preventing wound complications in kidney transplant recipients is needed to minimize morbidity and associated costs (2, 9, 11, 14). Preventive strategies include closed-incision negative pressure wound therapy (ciNPWT), “Prevena™” Incision Management system (KCI USA, Inc., San Antonio, TX, USA) (15). The WHO recommends using ciNPWT to prevent SSI in high-risk wounds (16). ciNPWT has been successfully utilized in other surgical disciplines including obstetrics (17, 18) and general surgery (19–21). However, there is a lack of evidence for the use of ciNPWT in transplant recipients, with only a single case report on the use of the Prevena™ dressing on a kidney transplant recipient being published (22). It is hypothesized that ciNPWT is associated with a reduction in wound complications in kidney transplantation.

## 2 Methods

### 2.1 Study design

This is a prospective observational cohort study. A total of 30 consecutive “high-risk” kidney transplant recipients were selected by the transplant surgeons to receive a ciNPWT dressing (intervention) over a 3-month period. The outcomes were compared to those of a non-study historical control group of subjects who received conventional hydrocolloid surgical dressing over a 12-month period. The study was conducted in a single-center tertiary referral transplant unit (Royal Prince Alfred Hospital Sydney, Australia) in 2018. A detailed methodology is provided in the thesis format of this study, and a study design diagram and flowchart are also presented in **Supplementary File 1** (23).

All subjects who received ciNPWT provided informed consent for participation in the study. This included information about the nature of the Prevena™ ciNPWT dressing (i.e., it differed from standard treatment), duration of treatment and follow-up, and complications associated with the device. Furthermore, a waiver of consent was obtained for the historical control group. Institutional ethics [Sydney Local Health District Human Research Ethics Committee (HREC/18/CRGH/127)] approval was obtained for this study, and the Prevena™ ciNPWT device had approval to be used in the hospital. This study conforms to the Declaration of Helsinki.

A total of 30 consecutive “high-risk” kidney transplant recipients were selected to receive a ciNPWT dressing if they satisfied the inclusion and exclusion criteria. The inclusion criteria for receiving a ciNPWT dressing were as follows: had a single kidney transplant with a closed incision and showed any of the following: body mass index (BMI) equal to or over 25 kg/m^2^, diabetes, previous history of abdominal surgery *via* the same incision (i.e., re-transplant), or any additional immunosuppression (defined as deviation from standard unit low- or intermediate-risk induction immunosuppression) (24). Patients were excluded from receiving a Prevena™ dressing if there was a contraindication to its use, including allergy to the dressing, inadequate wound hemostasis, or skin cellulitis prior to the application of ciNPWT. A 3-month period was chosen as a practical time frame to allow for consecutive recruitment and sufficient time for follow-up over 12 months, while the number of subjects (n = 30) was based on the number of Prevena™ dressings made available and donated in kind for the study by the company KCI-ACELITY, which was within the acceptable sample size limit for a pilot study

#### 2.1.1 Study treatment

Patients receiving a kidney transplant underwent general anesthesia. Prophylactic antibiotics [intravenous (IV) cephazolin] were given preoperatively within 2 h of the surgical incision. Patients were shaved with clippers as required. A three-way 18-Fr indwelling catheter was inserted and attached to the irrigation fluid bag. Aqueous or alcoholic betadine was used for skin preparation prior to draping. Standard protocol immunosuppression included induction with pulse methylprednisolone on days 0 and 1, followed by prednisolone 30 mg, and tapering to 10 mg by month 3. In addition, basiliximab and a tacrolimus with mycophenolate were given (25).

The standard approach to the retroperitoneum is *via* an oblique incision in the iliac fossa in this unit. End-to-side vascular anastomoses were performed for the renal artery and vein, and a modified Lich–Gregoir technique was used for the ureterocystostomy (26). Surgical drains were placed in the retroperitoneum at the discretion of the surgeon and were removed when the drain volume was <50 ml in 24 h. Fascial closure was performed in a continuous or interrupted fashion with 0-loop PDS^®^ or 0 nylon and the skin closed continuously with 4/0 Monocryl^®^ (poliglecaprone) (both from Ethicon, Johnson & Johnson Medical NV, Machelen, Belgium). Postoperative care consisted of a combined multidisciplinary medical, surgical, and nursing team approach on a specialized transplant ward according to unit protocol (25).

#### 2.1.2 Wound dressing

The conventional dressing is a hydrocolloid “Comfeel^®^ Plus” (Coloplast Pty Ltd., Victoria, Australia) dressing applied for 7 days. All patients were followed up over a 3-month period in the Outpatient Department by independent transplant staff. The wound outcomes were recorded in the clinical notes and maintained in a surgical outcomes REDCap database as routine practice.

**Figure 1:**
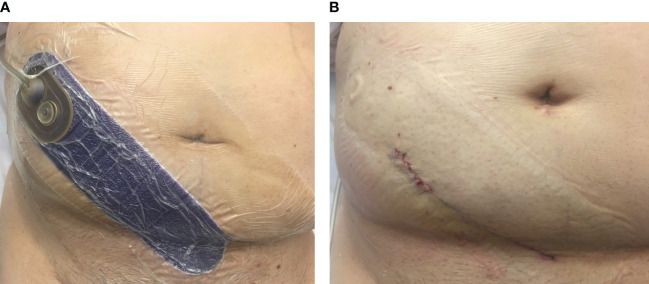
Clinical image of the closed-incision negative pressure wound therapy (ciNPWT) *in situ*. **(A)** “Prevena™” dressing applied to the kidney transplant incision in the right iliac fossa and connected to an integrated pump (not shown). **(B)** Wound appearance of the surgical incision after removal of ciNPWT (image obtained with patient consent).

The intervention group had a Prevena™ ciNPWT applied to the surgical site at the conclusion of the kidney transplant under sterile conditions in the operating theater (OT). The Prevena™ dressing is a polyurethane foam dressing lined with 0.019% silver ionic dressing and is placed over the surgical wound after skin closure. The dressing attaches to a battery-powered suction, connected to a 45-ml canister, and provides continuous negative pressure at −125 mmHg (**Figure 1A**). After 7 days of therapy, the dressings were removed and the wounds assessed (**Figure 1B**) and redressed for 7 days with a Comfeel^®^ dressing. Follow-up wound assessments were performed according to a standardized wound assessment form on days 14 and 30 and up to 3 months at the outpatient clinic by an independent, blinded assessor. Data were maintained in the REDCap database as part of the study.

#### 2.1.3 Outcomes

The primary composite outcome was the presence of wound complications within 3 months of transplantation. Secondary outcomes included SSI, wound dehiscence, return to the OT for wound-related reasons, the presence of a clinically significant hematoma or perigraft fluid collection (PFC) requiring intervention, use of open NPWT dressing for wound management, length of stay, and time to complete wound healing. The baseline characteristics of the recipient and donor and the surgical factors and complications were collected and reported.

#### 2.1.4 Definitions

Wounds were considered healed when the skin edge remained intact and approximated at the surgical incision site with no fluid drainage and no infection (27). In this study, a “wound complication” was defined as any SSI, wound dehiscence, perigraft lymphocele, or hematoma.

SSIs were defined according to the Australian Commission on Safety and Quality in Health Care guidelines (**Supplementary File 2**) (28), and wounds were assessed according to the “ASEPSIS” criteria developed by Wilson et al. (29) (**Supplementary File 3**).

### 2.2 Data collection and analysis

All data were collected and managed using a secure REDCap database (30). Comparisons were made between those who received conventional dressing and those who received ciNPWT dressing in the unmatched cohort. Analysis of an obese subset (BMI ≥30 kg/m^2^) and propensity score matching (PSM) were performed (see **Supplementary File 1** for the study design diagram and flowchart). Adverse events and deviations from the protocol were recorded. Missing data were noted and the data was analyzed accordingly. Continuous variables were presented as median with interquartile range, and the Mann–Whitney *U* test was used to compare non-normally distributed variables. Categorical variables were presented as number and percentage of the total and were compared using the *χ* ^2^ statistic. Fisher’s exact test was used where the cell counts were less than 5. Power and sample size estimations were also performed prior to the study with an online tool (clincalc.com) using existing data on high-risk wound complications of the lower abdomen (17), where the rate of surgical complications in the control group was 16% and that in the intervention (ciNPWT) group was 5%. At a significance level of 0.05 and at 80% power, an estimated 236 subjects would be required to test the efficacy of ciNPWT. The results of this study were used to calculate the number needed to treat (NNT) with ciNPWT in order to prevent a wound complication and to calculate the *post-hoc* power of the study (clincalc.com). Binary logistic regression was used to determine the predictors associated with wound complications. Collinearity and outliers were assessed. Statistical significance was set at *p* < 0.05, and all statistical analyses were performed using the Statistical Package for the Social Sciences software, version 25.0 (SPSS Inc., Chicago, IL, USA).

PSM was performed in order to reduce selection bias and balance the variables between the study groups (31). PSM analysis was performed, where the 97 subjects in the conventional dressing group were matched to 30 subjects in the ciNPWT group based on similar propensity scores. A logistic regression model was used to estimate the propensity scores, which predicted the probability of being assigned to either the conventional dressing or the ciNPWT group (32). The covariates used for calculating the propensity scores were chosen based on the potential to predict outcome and treatment assignment (33), which included age, obesity, diabetes, history of smoking, and the use of additional immunosuppression. The nearest neighbor 1:1 propensity matching without replacement was used. A caliper width of 0.2 of the standard deviation of the logit of the propensity score was used (34). Adequacy of matching was performed, indicating that matching improved the overall balance. Diagnostic plots were generated and confirmed adequate matching (see **Supplementary File 4**) (35).

## 3 Results

In the unmatched cohort, 30 kidney transplant recipients received the ciNPWT dressing; in the same calendar year, 97 kidney transplant recipients received conventional dressing. There were 127 subjects in total, all of whom had follow-up and were included in the analysis. **Table 1** summarizes the demographic data for the recipients and donors and the surgical factors in the unmatched cohort, obese subset, and PSM cohort.

**Table 1:** Demographic data and donor and surgical data of kidney transplant recipients who had conventional dressing or ciNPWT with unmatched, obese, and propensity score matching analyses.

Variable	Unmatched				BMI ≥30 kg/m^2^				PSM			
	Conventional dressing (*n* = 97)	ciNPWT/Prevena™ (*n* = 30)	Total (*n* = 127)	*p*-value	Conventional dressing (*n* = 19)	ciNPWT/Prevena™ (*n* = 16)	Total (*n* = 35)	*p*-value	Conventional dressing (*n* = 26)	ciNPWT/Prevena™ (*n* = 26)	Total (*n* = 52)	*p*-value
Male gender, n (%)	70 (72.2)	22 (73.3)	92 (72.4)	0.90	14 (73.7)	10 (62.5)	24 (68.6)	0.48	20 (76.9)	18 (69.2)	38 (73.1)	0.53
Age (years), median (IQR)	51.0 (39.5–60)	48.5 (39.5–61.2)		0.90	55 (46–65)	47 (41 –61)		0.30	50.0 (42–58.7)	47 (39.5–61.25)		0.72
CCI, median (IQR)	2.0 (2–4)	2.5 (2–4)		0.62	4 (2–5)	3.5 (2–4.7)		0.86	3 (2–4.2)	2 (2–4)		0.26
Diabetes, *n* (%)	18 (18.6)	9 (30)	27 (21.3)	0.18	8 (42.1)	5 (31.3)	13 (37.1)	0.51	8 (30.8)	7 (26.9)	15 (28.8)	0.76
Ever smoked, *n* (%)	30 (30.9)	12 (40)	42 (33.1)	0.35	8 (42.1)	5 (31.1)	13 (37.1)	0.51	11 (42.3)	10 (38.5)	21 (40.4)	0.77
BMI, median (IQR)	25.5 (22.4–29.0)	30.0 (26.6–32.5)		<0.001*	32.6 (31.1–34.9)	32.5 (30.9–35.5)		0.68	28.6 (25–32.8)	29.3 (26.4–32.5)		0.67
BMI categories, *n* (%)				0.004*				0.54				0.67
<18.5 underweight	2 (2.1)	0 (0)	2 (1.6)						0 (0)	0 (0)	0 (0)	
18.5 – 24.9 healthy	41 (42.3)	4 (13.3)^a^	45 (35.4)						6 (23.1)	4 (15.4)	10 (19.2)	
25– 29.9 overweight	35 (36.1)	10 (33.3)	45 (35.4)						8 (30.8)	10 (38.5)	18 (34.6)	
30–34.9 obese I	15 (15.5)	12 (40.0)^a^	27 (21.3)		15 (78.9)	12 (75)	27 (77.1)		9 (34.6)	8 (30.8)	17 (32.7)	
35–39.9 obese II	3 (3.1)	4 (13.3)^a^	7 (5.5)		3 (15.8)	4 (25)	7 (20)		2 (7.7)	4 (15.4)	6 (11.5)	
>40 obese III	1 (1.0)	0 (0)	1 (0.8)		1 (5.3)	0 (0)	1 (2.9)		1 (3.8)	0 (0)	1 (1.9)	
Cause of renal failure, *n* (%)				0.38				0.22				0.27
DM	13 (13.4)	5 (16.7)	18 (14.2)		8 (42.1)	3 (18.8)	11 (31.4)		6 (23.1)	4 (15.4)	10 (19.2)	
GN	11 (11.3)	4 (13.3)	15 (11.8)		1 (5.3)	2 (12.5)	3 (8.6)		6 (23.1)	4 (15.4)	10 (19.2)	
HTN	8 (8.2)	2 (6.7)	10 (7.9)		2 (10.5)	2 (12.5)	4 (11.4)		4 (15.4)	2 (7.7)	6 (11.5)	
IgA	10 (10.3)	4 (13.3)	14 (11)		2 (10.5)	2 (12.5)	4 (11.4)		3 (11.5)	4 (15.4)	7 (13.5)	
PCKD	14 (14.4)	1 (3.3)	15 (11.8)		3(15.8)	0 (0)	3 (8.6)		2 (7.7)	1 (3.8)	3 (5.8)	
Reflux	10 (10.3)	0 (0)	10 (7.9)						2 (7.7)	0 (0)	2 (3.8)	
Autoimmune	2 (2.1)	1 (3.3)	3 (2.4)						0 (0)	1 (3.8)	1 (1.9)	
Other	29 (29.9)	13 (43.3)	42 (33.1)		3 (15.8)	7 (43.8)	10 (28.6)		3 (11.5)	10 (38.5)^a^	13 (25)	
Dialysis	87 (89.7)	26 (86.7)	113 (89)	0.64	18 (94.7)	14 (87.5)	32 (91.4)	0.58	24 (92.3)	24 (92.3)	48 (92.3)	
Type of dialysis, *n* (%)				0.67				1.00				0.50
HD	60 (69)	20 (76.9)	80 (70.8)		14 (77.8)	11 (78.6)	25 (78.1)		17 (70.8)	19 (79.2)	36 (75)	
PD	26 (29.9)	6 (23.1)	32 (28.3)		4 (22.2)	3 (21.4)	7 (21.9)		7 (29.2)	5 (20.8)	12 (25)	
Vascath	1 (1.1)	0 (0)	1 (0.9)									
Days dialysis, median (IQR)	1,176 (749–1,749)	1,173 (505–2,398)		0.92	1,477 (963–2,398)	1,520 (505–3,779)		0.69	1,693 (941–3,697)	1,188 (505–3,779)		0.80
First transplant, *n* (%)	88 (90.7)	27 (90)	115 (90.6)	1.00	18 (94.7)	14 (87.5)		0.58	24 (92.3)	23 (88.5)	47 (90.4)	1.00
Living donor, *n* (%)	23 (23.7)	14 (46.7)^a^	37 (29.1)	0.02*	2 (10.5)	6 (37.5)	8 (22.9)	0.10	7 (26.9)	11 (42.3)	18 (34.6)	0.24
Deceased donor, *n* (%)	74 (76.3)	16 (53.3)^a^	90 (70.9)		17 (89.5)	10 (62.5)	27 (77.1)		19 (73.1)	15 (57.7)	34 (65.4)	
DBD	54 (73)	11 (68.8)	65 (72.2)	0.76	15 (88.2)	6 (60)	21 (77.8)	0.09	14 (73.7)	11 (73.3)	25 (73.5)	1.00
DCD	20 (27)	5 (31.3)	25 (27.8)		2 (11.8)	4 (40)	6 (22.2)		5 (26.3)	4 (26.7)	9 (26.5)	
Donor BMI, median (IQR)	26.2 (22.9–29.8)	26.4 (23.7–31.2)		0.35	25.5 (23–27.8)	25.7 (22.3–31.1)		0.76	26.6 (23.8–31.0)	26.4 (23.7–31.2)		0.90
CIT (min), median (IQR)	456 (177–702)	386 (159–671)		0.34	659 (366–822)	484 (147–673)		0.20	450 (189–716)	397 (164–670)		0.79
WIT (min), median (IQR)	32 (22–40)	33 (23–42)		0.84	30 (23–36)	33 (23–43)		0.35	31 (23–41)	35 (24 –42)		0.58
Surgical drain, *n* (%)	27 (27.8)	9 (30)	36 (28.3)	0.74	3 (15.8)	6 (37.5)	9 (25.7)	0.25	6 (23.1)	7 (26.9)	13 (25)	0.75

For categorical variables where the cell count is <5, p-values were obtained with the Fisher’s exact test.

BMI, body mass index; ciNPWT, closed-incision negative pressure wound therapy; CCI, Charlson comorbidity index; Cont., continuous; DM, diabetes mellitus; DBD, donation after brain death; DCD, donation after circulatory death; GN, glomerulonephritis; HD, hemodialysis; HTN, hypertension; IgA, immunoglobulin A; IQR, interquartile range; PCKD, polycystic kidney disease; PD, peritoneal dialysis; PSM, propensity score matching; SD, standard deviation; Vascath, vascular catheter; WIT, warm ischemic time.

*p < 0.05.

a Difference in proportions between columns.

The two groups were similar across most baseline characteristics in the unmatched cohort (**Table 1**); however, the median recipient BMI was 30 kg/m^2^ (range, 26.6–32.5 kg/m^2^) in the ciNPWT group, which was higher compared to the 25.5 kg/m^2^ (range, 22.4–29.0 kg/m^2^) in the conventional dressing group (*p* < 0.001). In the ciNPWT group, there were more living donors compared with the conventional dressing group [46.7% (14/30) and 23.7% (23/97), respectively, *p* = 0.02].

The outcomes and complications for all kidney transplant recipients and the subset analyses are shown in **Table 2**. The primary study outcome of wound complications occurred in 21.3% (27/127) of all subjects overall. There was no significant difference in the wound complication rates between the ciNPWT (23.3%, 7/30) and conventional dressing (20.6%, 20/97) groups (*p* = 0.75). For secondary outcomes, the overall rate of SSI was 7.1% (9/127). The SSI rate was 13.3% (4/30) in the ciNPWT group and was 5.2% (5/97) in the conventional dressing group, which was not significantly different (*p* = 0.21). The overall wound dehiscence rate was 14.2% (18/127), of which 20% (6/30) occurred in the ciNPWT group and 12.4% (12/97) in the conventional dressing group, although the difference was not significant (*p* = 0.29) (**Table 2**). Deep fascial dehiscence occurred in three (3.1%) subjects in the conventional dressing group only, two of whom suffered concomitant deep SSI. The rates of return to OT for wound complications, intervention for PFC, hematoma, use of open NPWT, time to wound healing, and median length of stay were not different between the groups (**Table 2**). The median ASPESIS score was 15.5 (IQR = 4.2–30) in the ciNPWT group, which was higher compared to the ASEPSIS score of 0 (IQR = 0–5.5) in the conventional dressing group (*p* = 0.02).

**Table 2:** Outcomes and complications in kidney transplant recipients who had conventional dressing or ciNPWT with unmatched, obese, and propensity score matching analyses.

Variable	Unmatched				BMI ≥30 kg/m^2^					PSM				
	Conventional dressing (*n* = 97)	ciNPWT/Prevena™ (*n* = 30)	Total (*n* = 127)	*p*-value	Conventional dressing (*n* = 19)	ciNPWT/Prevena™ (*n* = 16)	Total (*n* = 35)	*p*-value		Conventional dressing (*n* = 26)	ciNPWT/Prevena™ (*n* = 26)	Total (*n* = 52)	*p*-value	
Primary outcome														
Wound complication, *n* (%)	20 (20.6)	7 (23.3)	27 (21.3)	0.75	7 (36.8)	5 (31.3)	12 (34.3)	0.73		6 (23.1)	6 (23.1)	12 (23.1)	1.00	
Secondary outcomes														
Surgical site infection, *n* (%)	5 (5.2)	4 (13.3)	9 (7.1)	0.21	3 (15.8)	3 (18.8)	6 (17.1)		1.00	2 (7.7)	3 (11.5)	5 (9.6)		1.00
Wound dehiscence, *n* (%)	12 (12.4)	6 (20)	18 (14.2)	0.29	6 (31.6)	4 (25)	10 (28.6)		0.72	5 (19.2)	5 (19.2)	10 (19.2)		
Superficial dehisc., *n* (%)	9 (9.3)	6 (20)	15 (11.8)	0.19	3 (15.8)	4 (25)	7 (20)		0.23	3 (11.5)	5 (19.2)	8 (15.4)		0.28
Deep fascial dehisc., *n* (%)	3 (3.1)	0 (0)	3 (2.4)		3 (15.8)	0 (0)	3 (8.6)			2 (7.7)	0 (0)	2 (3.8)		
Return to OT (wound), *n* (%)	5 (5.2)	3 (10)	8 (6.3)	0.39	3 (15.8)	2 (12.5)	5 (14.3)		1.00	2 (7.7)	2 (7.7)	4 (7.7)		1.00
PFC treatment, *n* (%)	7 (7.2)	0 (0)	7 (5.5)	0.20	1 (5.3)	0 (0)	1 (2.9)		1.00	1 (3.8)	0 (0)	1 (1.9)		1.00
Hematoma, *n* (%)	4 (4.1)	3 (10)	7 (5.5)	0.35	1 (5.3)	1 (6.3)	2 (5.7)		1.00	1 (3.8)	2 (7.7)	3 (5.8)		1.00
Use of NPWT, *n* (%)	8 (8.2)	3 (10)	11 (8.7)	0.72	4 (21.1)	1 (6.3)	5 (14.3)		0.35	4 (15.4)	3 (11.5)	7 (13.5)		1.00
Time to wound healing (days), median (IQR)	89 (28–126)	53 (30–72)		0.28	129 (115–164)	54 (41–64)			0.03*	101 (76–150)	53 (30–72)			0.20
Length of stay, median (IQR)	7 (6–12)	7 (6–14)		0.78	7 (7–12)	6.5 (6–14.7)			0.78	9 (7–13)	7 (6–12)			0.20
ASEPSIS score, median (IQR)	0 (0–5.5)	15.5 (4.2–30)		0.02*	16 (1.0–52.5)	20.5 (5.7–42)			0.99	4 (0–33)	14 (3.5–36)			0.55
Graft loss, *n* (%)	2 (2.1)	1 (3.4)	3 (2.4)	0.56	0 (0)	1 (6.3)	1 (2.9)		0.46	0 (0)	0 (0)	0 (0)		
DGF, *n* (%)	31 (32)	11 (36.7)	42 (33.1)	0.63	8 (42.1)	7 (43.8)	15 (42.9)		0.92	10 (38.5)	10 (38.5)	20 (38.5)		1.00
Extra immunosuppress., *n* (%)	24 (24.7)	11 (36.7)	35 (27.6)	0.20	3 (15.8)	6 (37.5)	9 (25.7)		0.14	8 (30.8)	8 (30.8)	16 (30.8)		1.00
Drain days, median (IQR)	5 (3–16)	4 (3–12)		0.95	8 (5–10)	4 (4–12)			0.99	4 (2–10)	7 (3–13)			0.81
Follow-up days, median (IQR)	78 (58–140)	85 (70–129)		0.82	103 (55–187)	90 (55–156)			0.75	63 (48–192)	85 (71–115)			0.47

For categorical variables where the cell count was <5, p-values were obtained with Fisher’s Exact test.

BMI, body mass index; ciNPWT, closed-incision negative pressure wound therapy; Dehisc., dehiscence; DGF, delayed graft function; immunosuppress., immunosuppression; NPWT, negative pressure wound therapy; OT, operating theater; PFC, perigraft fluid collection; PSM, propensity score matching.

*p < 0.05.

There was one death, as well as three recipients with graft loss over the 3-month postoperative period (**Table 2**). There was no difference in DGF or the rates of additional immunosuppression between the dressing types. Median follow-up times were similar, 85 days (IQR = 70–129 days) and 78 days (IQR = 58–140 days) for those in the ciNPWT and conventional dressing groups, respectively (*p* = 0.82) (**Table 2**).

On univariable analysis, the variables age, obesity, and male gender were correlated with wound complications, but the dressing type was not (**Table 3**). On multivariable analysis, increased age was associated with decreased odds of wound complications [odds ratio (OR) = 0.95, 95% confidence interval (CI) = 0.92–0.99, *p* = 0.017], while obesity was associated with increased odds of wound complications (OR = 4.27, 95% CI = 1.44–12.69, *p* = 0.009).

**Table 3:** Univariable and multivariable logistic regression for wound complications.

Wound complication	Univariable		Multivariable	
Variable	OR (95% CI)	*p*-value	OR (95% CI)	*p*-value
Type of dressing	1.17 (0.44–3.08)	0.75	0.68 (0.21–2.15)	0.50
Age	0.96 (0.93–0.99)	0.03*	0.95 (0.92–0.99)	0.017*
Obesity	2.68 (1.10–6.53)	0.03*	4.27 (1.44–12.69)	0.009*
Male gender	2.58 (2.49–2.68)	<0.001*	3.04 (0.92–10.11)	0.07
Additional immunosuppression	1.76 (0.71–4.35)	0.22	1.1 (.55–4.14)	0.42
WIT	0.99 (0.95–1.03)	0.48	0.99 (0.95–1.03)	0.48
Type of donor	0.78 (0.31–1.93)	0.59		
Smoking history	1.53 (0.64–3.68)	0.34		
Drain	1.14 (0.45–2.93)	0.78		
Diabetes	1.4 (0.52–3.77)	0.51		
DGF	0.65 (0.25–1.69)	0.38		

DGF, delayed graft function; WIT, warm ischemic time.

*p < 0.05.

### 3.1 Obese (BMI ≥30 kg/m^2^) subset

In total, there were 35 subjects with obesity, 16 in the ciNPWT group and 19 in the conventional dressing group. The baseline characteristics are presented in **Table 1**. The median BMI was 32.5 kg/m^2^ (IQR = 30.9–35.5 kg/m^2^) in the ciNPWT group and 32.6 kg/m^2^ (IQR = 31.1–34.9 kg/m^2^) in the conventional dressing group (*p* = 0.68). Wound complications occurred in 34.3% (12/35) of all subjects with obesity (**Table 2**). There were fewer wound complications in the ciNPWT group (31.3%, 5/16) compared with the conventional dressing group (36.8%, 7/19), although not significant (*p* = 0.73). Thus, based on these values, the NNT in order to prevent a wound complication in kidney transplant recipients with obesity was 18.

For the obese subset, the overall SSI rate was 17.1% (6/35), while the wound dehiscence rate was 28.6% (10/35), with no significant difference between the conventional and ciNPWT groups (**Table 2**). Three deep fascial dehiscence occurred in subjects with obesity who had conventional dressings only. Moreover, there was no difference in other secondary outcomes including intervention for PFC, hematoma, use of open NPWT, and return to OT (**Table 2**). The median time to wound healing in the obese subset was 54 days (IQR = 41–64 days) in the ciNPWT group and 129 days (IQR = 115–164 days) in the conventional dressing group, which was significantly different (*p* = 0.03). The median ASEPSIS scores were 20.5 (IQR = 5.7–42) and 16 (IQR = 1.0–52.5) in the ciNPWT and conventional dressing groups, respectively, which were not significantly different (*p* = 0.99) (**Table 2**).

### 3.2 Propensity score matching analysis

PSM generated 26 matched pairs in each group, with a total of 52 subjects. The baseline characteristics (**Table 1**), including BMI, were well matched between the conventional and ciNPWT groups [28.6 kg/m^2^ (IQR = 25.0–32.8 kg/m^2^) and 29.3 kg/m^2^ (IQR = 26.4–32.5 kg/m^2^), respectively, *p* = 0.67] (**Table 1**). The overall wound complications rate was 23.1% (12/52) and was equivalent in the conventional dressing and ciNPWT groups (**Table 2**). The overall SSI rate was 9.6% (5/52), while the overall wound dehiscence rate was 19.2% (10/52), with no difference between the dressing types for the other outcomes (**Table 2**).

### 3.3 Adverse events and deviations from protocol

Minor adverse events included small skin tears from the plastic drape of the ciNPWT in two subjects overall. Deviations from protocol occurred in five subjects. A total of three patients required a return to the OT for graft exploration and early removal of the ciNPWT dressing; of these patients, two developed small superficial wound dehiscence following primary re-closure. The third case developed a device malfunction from the overfilling of the 45-ml canister and was switched to low wall suction. This subject had a small hematoma, which was managed conservatively. The fourth case was non-compliant with wound management and subsequently developed superficial wound dehiscence and SSI, resulting in surgical debridement and open NPWT.

## 4 Discussion

Wound complications are a common cause of morbidity after kidney transplantation, particularly given the combination of a large lower abdominal incision and risk factors including immunosuppression, overweight/obesity, and diabetes (6, 36). Wound complications can be resource-intensive and complex to manage (11, 13, 37). Strategies to reduce and minimize wound complications are thus required to decrease any associated morbidity in kidney transplant recipients, which was the rationale behind evaluating the closed-incision negative pressure wound management system in this study.

This study reported on the use of a closed-incision negative pressure wound dressing in 30 kidney transplant recipients. The results did not demonstrate an improvement in the primary outcome of wound complications with the use of the ciNPWT dressing compared with conventional dressing in 97 subjects [23.3% (7/30) and 20.6% (20/97), respectively, *p* = 0.75]. After PSM, the proportion of recipients with obesity was balanced, and the 23.1% (6/26) rate of wound complications was equivalent in both groups, indicating that there was no benefit of the ciNPWT when taking into account all subjects and with balanced covariates. Further analysis in the obese subset (BMI ≥30 kg/m^2^) showed a reduction in wound complications with the use of the ciNPWT dressing compared to conventional dressing; however, it was not significantly different [31.1% (5/16) and 36.8% (7/19), respectively, *p* = 0.73], and the NNT to prevent one wound complication in an obese transplant recipient was 18.

The other outcomes including subcategories of SSI and wound dehiscence, return to OT for wound-related reasons, hematoma, use of open NPWT dressing for wound management, PFC treatment, length of stay, readmission to hospital, and time to complete wound healing were not significantly improved with the use of ciNPWT (**Table 2**).

While most baseline characteristics were similar across the dressing types, in the unmatched analysis, there was a significantly lower median BMI in the conventional dressing group (25.5 kg/m^2^, IQR = 22.4–29 kg/m^2^) compared with the ciNPWT group (30 kg/m^2^, IQR = 26.6–32.5 kg/m^2^, *p* < 0.001). This imbalance was likely due to the selection of high-risk patients, including those overweight and obese, which is strongly associated with wound complications (2, 4, 11, 38, 39). Given the potential for a selection bias of high-risk kidney transplant patients receiving ciNPWT, a significantly higher wound complication rate in this group was anticipated; however, there was no difference demonstrated between groups. This may indicate some benefit of the ciNPWT; however, given the small sample size, the results in this study may therefore be subject to a type II error.

The overall wound complication rate in this study was 21.3% (27/127), which was higher than the previously reported 30-day abdominal wall complication rate of 7.7% (64/828) in our unit (7). This may be due to the wider definition of wound complications used in this study. The overall rate of SSI was 7.1% (9/127), and it was 17.1% (6/35) in the obese subset (**Table 3**). These rates are consistent with those in the literature (2, 40). Open NPWT was utilized in 11 of the 27 (40.7%) patients with wound complications, which was higher than the previously reported 27% (17/64) in our unit (7). This reflects a change in wound management, with more expertise and preference for using NPWT over time.

In the analysis of the obese subset, 3 patients with conventional dressings developed deep fascial dehiscence and 2 had concomitant SSI, requiring reoperative surgery. Furthermore, in this subset, the median time to wound healing was lower in the ciNPWT group when compared with the conventional dressing group [54 days (IQR = 41–64) and 129 days (IQR = 115–164), respectively, *p* = 0.03] despite similar ASEPSIS scores (**Table 2**). However, this was no longer significant after PSM, suggesting the presence of confounding factors. The ciNPWT apparently redistributes lateral tensile strength at the superficial and deep suture lines and thus promotes wound healing of the skin and subcutaneous space (41). However, it is unclear how well ciNPWT can mitigate the effects of obesity, such as the traction effect of the abdominal pannus and tension across the transplant incision (11). Furthermore, ciNPWT may mitigate the effect of wound complications in certain conditions, although this remains unclear (41).

The type of dressing used was not associated with wound complications in the univariable analysis (**Table 3**), nor were surgical drains, diabetes, additional immunosuppression, donor type, or smoking. The multivariable analysis showed that obesity was associated with increased odds of wound complications (OR = 4.27, 95% CI = 1.44–12.69, *p* = 0.009), which is in keeping with the known risk factors for wound complications.

Evidence for the use of ciNPWT in kidney transplant recipients is lacking. The findings in our study are consistent with reports from other surgical specialties in the literature. A randomized control trial of 2,035 obese, high-risk women undergoing cesarean section demonstrated a 3% reduction in the absolute risk of SSI with ciNPWT compared to standard dressing, which was close to significance on per intention-to-treat analysis (*p* = 0.06) (42). While a Cochrane review indicated a probable reduction in SSI with ciNPWT compared with standard dressing [8.8% and 13%, respectively; relative risk (RR) = 0.66, 95% CI = 0.55–0.80, *I* ^2 =^ 23%], there was no clear difference in the wound dehiscence rates (5.3% and 6.2%, respectively; RR = 0.88, 95% CI = 0.69–1.13, *I* ^2 =^ 0%) and no effect on seroma or the reoperation rates (43). Some of these results differed from those of our study, which could be due to differences in the definitions of “high risk,” wound type, comorbidity profiles, and the intrinsic differences in healthcare settings.

The reported adverse events from ciNPWT use included skin blistering, which has restricted its use in orthopedic wounds until safety can be established (44). In our study, ciNPWT was well tolerated overall. While two patients suffered small skin tears, this was managed conservatively. Given that there were no significant adverse events, our study does support the safety of this dressing in kidney transplant recipients.

The strengths of this study are as follows: 1) it is the largest reported study evaluating the use of the Prevena™ ciNPWT in kidney transplant recipients; 2) the use of contemporaneous intervention and control groups, which reduced the potential for an era effect on the outcomes; 3) complete follow-up for the majority of patients; and 4) and only a small number of patients violated the study protocol.

The limitations of this study include the study design, given it is a non-randomized and non-blinded cohort, in addition to the small sample size. The sample size of 30 was determined by the number of ciNPWT dressings made available for the study by KCI-Acelity. Although within the required sample size limit for a pilot study, this made it an underpowered study, with *post-hoc* power calculation of 5.4% for the primary outcome of wound complications. Subset analysis was conducted to account for the imbalance in recipients with obesity in the intervention group. Furthermore, PSM was conducted to address the confounding factors and selection bias. Potential weaknesses include the recruitment and selection bias, a reporting bias or “Hawthorne effect” that describes changes in behavior as a result of being observed (45); however, blinding of the wound assessment for the ciNPWT group aimed to minimize this. Additional limitations include the different data handling and follow-up for the historical control group, although the end outcomes of all surgical outcomes, including wound complications, were routinely recorded for all transplant recipients.

One disadvantage of the Prevena™ ciNPWT dressing is its cost, i.e., AUD $295 per device. Furthermore, Prevena™ is single-use only, and early removal renders the dressing unusable. Future studies should include an economic analysis.

This study indicated no significant difference in the use of the Prevena™ ciNPWT dressing in reducing wound complications in kidney transplant recipients. However, this study provided information on the wound complication rates for kidney transplant recipients in the current era, particularly those who were obese and high-risk at our center. Furthermore, the findings from this study could be useful for informing future studies, including the protocol development for a randomized control trial (Trial ID: NCT03948412). Future research should include comparing different commercially available ciNPWT devices and the identification a subgroup of high-risk renal transplant recipients, such as those with obesity, and tailoring wound care.

## 5 Conclusion

This is the first reported cohort study evaluating the use of ciNPWT in kidney transplantation. While ciNPWT is safe and was well tolerated, it was not associated with a statistically significant reduction in wound complications when compared to conventional dressing. The findings from this study will be used to inform future studies associated with the use of ciNPWT in kidney transplantation.

## Data availability statement

The raw data supporting the conclusions of this article will be made available by the authors, without undue reservation.

## Ethics statement

The studies involving humans were approved by the Sydney Local Health District Human Research Ethics Committee (HREC/18/CRGH/127). The studies were conducted in accordance with the local legislation and institutional requirements. The participants provided their written informed consent to participate in this study. Written informed consent was obtained from the individual(s) for the publication of any potentially identifiable images or data included in this article.

## Author contributions

SL: Conceptualization, Data curation, Formal analysis, Investigation, Methodology, Project administration, Writing – original draft, Writing – review & editing. AH: Data curation, Writing – original draft, Writing – review & editing. TY: Writing – original draft, Writing – review & editing. CS: Conceptualization, Writing – original draft, Writing – review & editing. DG: Writing – original draft, Writing – review & editing. HP: Writing – original draft, Writing – review & editing. SC: Writing – original draft, Writing – review & editing, Conceptualization. JL: Conceptualization, Formal analysis, Supervision, Writing – original draft, Writing – review & editing.
