# Supplementary material for: Prospective evaluation of a closed-incision negative pressure wound therapy system in kidney transplantation and its association with wound complications
Source: Front Nephrol. 2024 Feb 27;4:1352363. doi: 10.3389/fneph.2024.1352363 (PMC10929013; doi:10.3389/fneph.2024.1352363)
Supplement: Supplementary file 1 [file DataSheet_1.docx]

Supplementary Material

# Supplementary Data

**Supplementary file 1: Study design diagram and flow chart**

**Figure 1a.** Study Design diagram with obese subset analysis

##

**Figure 1b**. Study Design diagram with Propensity Score Matched analysis

**Supplementary file 2: Surgical Site Infection (SSI) definition**

The Australian Commission of Quality in Health Care (ACQHC) has the following definitions for SSI (28):

A superficial incisional SSI must meet the following criteria:

occur within 30 days after an operation, involve only the skin and subcutaneous tissue of the incision and the patient must have at least one of the following:

1. purulent drainage from the superficial incision
2. organisms isolated from an aseptically collected culture of fluid or tissue
3. the patient has at the site of infection any of the signs and symptoms including:

- pain or tenderness, localised swelling, redness or heat, and the incision is explored by the surgeon

1. diagnosis of superficial SSI by surgeon

A deep incisional or organ /space SSI must meet the following criteria:

occur within 30 days after the operation, involves deep soft tissues of the incision, and the patient has at least one of the following

1. purulent drainage from the deep soft tissue or drain that is placed through a stab wound into the organ/space
2. a deep incision that spontaneously dehisces, or deliberately opened by a surgeon and the patient has at least one of the signs or symptoms of:
   1. Fever (> 38^0^ C), localized pain or tenderness with culture positive specimen
   2. Organisms isolated from aseptically obtained culture of fluid or tissue
   3. An abscess or other evidence of infection involving the deep incision
   4. Diagnosis of, or antimicrobial treatment of a deep incision or organ/space SSI by the surgeon

**Supplementary file 3: ASEPSIS Criteria**

ASEPSIS Criteria developed by Wilson et al (29) and scores surgical site infections against a criteria of ‘**A**dditional treatment’ ‘**S**erous Discharge’, **E**rythema, **P**urulent Exudate, **S**eparation of deep tissues, **I**solation of bacteria, and **S**tay as inpatient prolonged (> 14 day).

An ASEPSIS score of:

0 -10 indicates satisfactory healing

11-20 indicates a disturbance of healing

21 -30 indicates minor wound healing

31 -40 indicates a moderate wound infection

> 40 indicates a severe wound infection

**Supplementary file 4: Propensity Score Matching Diagnostic Plots**


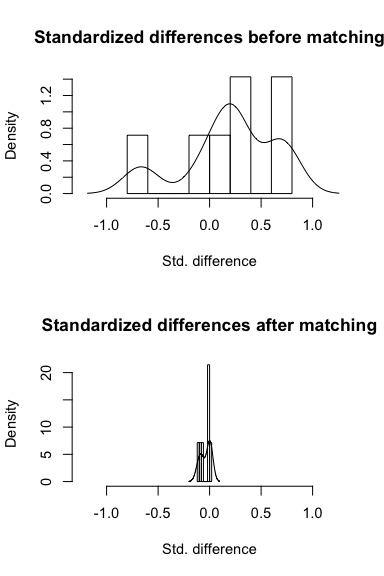

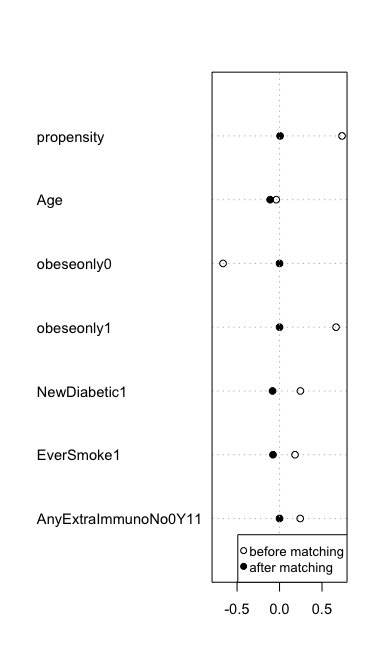


b

a

**Figure 4a**. Standardized differences before and after propensity score matching displayed as a histogram with overlaid kernel density estimates, indicating improved covariate balance after matching b) standardized mean differences displayed for covariates as dot plot form, indicating balance amongst covariates after matching


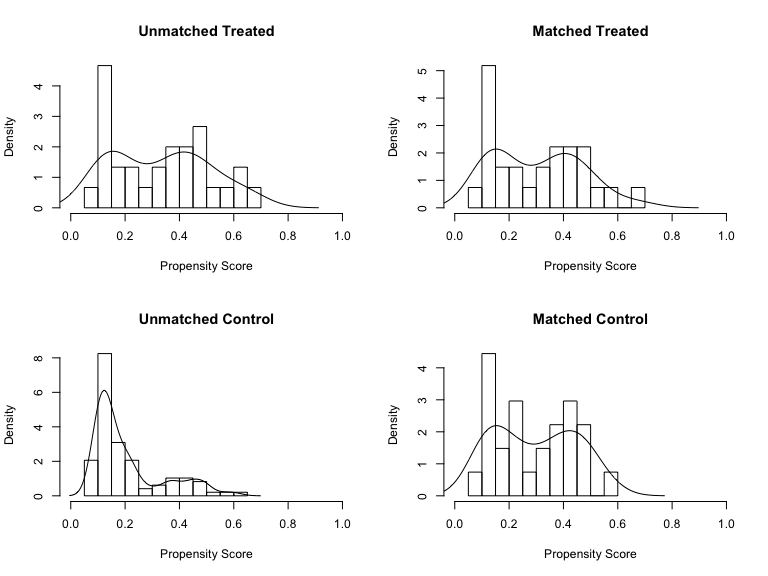


**Figure 4b.** Histogram with overlaid kernel density estimates for propensity score for unmatched and matched treatment and control groups, indicating improved covariate balance after matching.


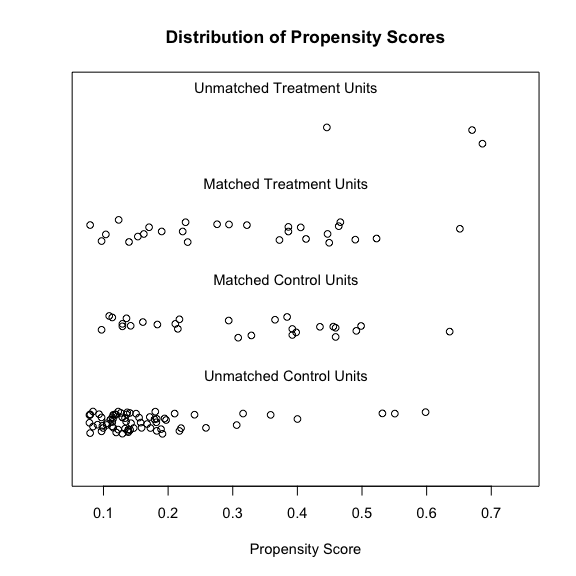


**Figure 4c**. Distribution of propensity score for individual units and if they are matched or discarded in treatment and control groups.
